# Ion-specific nanoscale compaction of cysteine-modified poly(acrylic acid) brushes revealed by 3D scanning force microscopy with frequency modulation detection†

**DOI:** 10.1039/d2na00350c

**Published:** 2022-09-15

**Authors:** Akihisa Yamamoto, Takahiko Ikarashi, Takeshi Fukuma, Ryo Suzuki, Masaki Nakahata, Kazuki Miyata, Motomu Tanaka

**Affiliations:** Center for Integrative Medicine and Physics, Institute for Advanced Study, Kyoto University Kyoto 606-8501 Japan yamamoto.akihisa.6w@kyoto-u.ac.jp; Division of Nano Life Science, Kanazawa University Kanazawa 920-1192 Japan k-miyata@staff.kanazawa-u.ac.jp; Nano Life Science Institute (WPI-NanoLSI), Kanazawa University Kanazawa 920-1192 Japan; Department of Materials Engineering Science, Graduate School of Engineering Science, Osaka University Osaka 560-8531 Japan; Department of Macromolecular Science, Graduate School of Science, Osaka University Osaka 560-0043 Japan; Physical Chemistry of Biosystems, Institute of Physical Chemistry, Heidelberg University 69120 Heidelberg Germany tanaka@uni-heidelberg.de

## Abstract

Stimuli-responsive polyelectrolyte brushes adapt their physico-chemical properties according to pH and ion concentrations of the solution in contact. We synthesized a poly(acrylic acid) bearing cysteine residues at side chains and a lipid head group at the terminal, and incorporated them into a phospholipid monolayer deposited on a hydrophobic silane monolayer. The ion-specific, nanoscale response of polyelectrolyte brushes was detected by using three-dimensional scanning force microscopy (3D-SFM) combined with frequency modulation detection. The obtained topographic and mechanical landscapes indicated that the brushes were uniformly stretched, undergoing a gradual transition from the brush to the bulk electrolyte in the absence of divalent cations. When 1 mM calcium ions were added, the brushes were uniformly compacted, exhibiting a sharper brush-to-bulk transition. Remarkably, the addition of 1 mM cadmium ions made the brush surface significantly rough and the mechanical landscape highly heterogeneous. Currently, cadmium-specific nanoscale compaction of the brushes is attributed to the coordination of thiol and carboxyl side chains with cadmium ions, as suggested for naturally occurring, heavy metal binding proteins.

## Abstract 2

Using three-dimensional scanning force microscopy (3D-SFM) combined with frequency modulation detection, we detected the cadmium ion-specific nanoscale compaction of poly(acrylic acid) brushes modified with cysteine.

## Graphical abstract

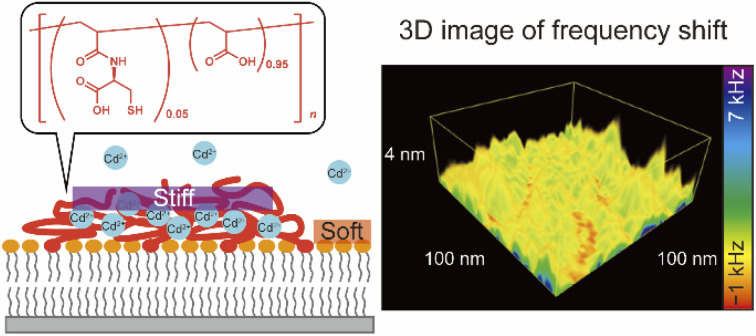

## Introduction

Stimuli-responsive polymers have attracted interest for use in various drug delivery and microencapsulation applications because these substances can protect and/or release materials in response to the surrounding environment.^1^ In particular, polyelectrolyte brushes have been grafted on surfaces to give the materials adaptable functionalities. In these cases, the physical properties of the surface can be modulated based on the electrostatic properties of the external media (*e.g.*, pH and salt concentrations).^2–4^ The structures and mechanical properties of polymer brushes have been measured experimentally using X-ray/neutron reflectivity,^5,6^ quartz crystal microbalance with dissipation (QCM-D),^7,8^ tribology,^9,10^ and microinterferometry.^11,12^

Among the various techniques, atomic force microscopy (AFM) is commonly used to investigate the mechanical properties of polymer brushes *via* nanoindentation.^13,14^ Recently, Fukuma *et al.*, developed three-dimensional scanning force microscopy (3D-SFM) based on frequency modulation AFM (FM-AFM),^15^ which has been utilized to construct force maps of material surfaces. During the measurement process, a cantilever tip scans in the vicinity of the interface both parallel and perpendicular to the surface, and the frequency shift of the oscillating cantilever is recorded in 3D space. This technique has been successful in visualizing the 3D force maps of the surfaces of various materials in water, *e.g.*, minerals,^16,17^ graphene,^18^ graphite,^19^ and supported phospholipid bilayers.^20^ A key advantage of this technique is that it can be used to investigate the density distribution of water at the solid/water interface. By exploiting this unique functionality, 3D-SFM has been applied to observe the ammonia-mediated hydration of poly(vinyl alcohol) coated surfaces.^21^ However, to our knowledge, no experimental studies have demonstrated the potential of 3D-SFM to detect dynamic modulations of hydrated polymer brushes driven by external chemical stimuli.

**Fig. 1:**
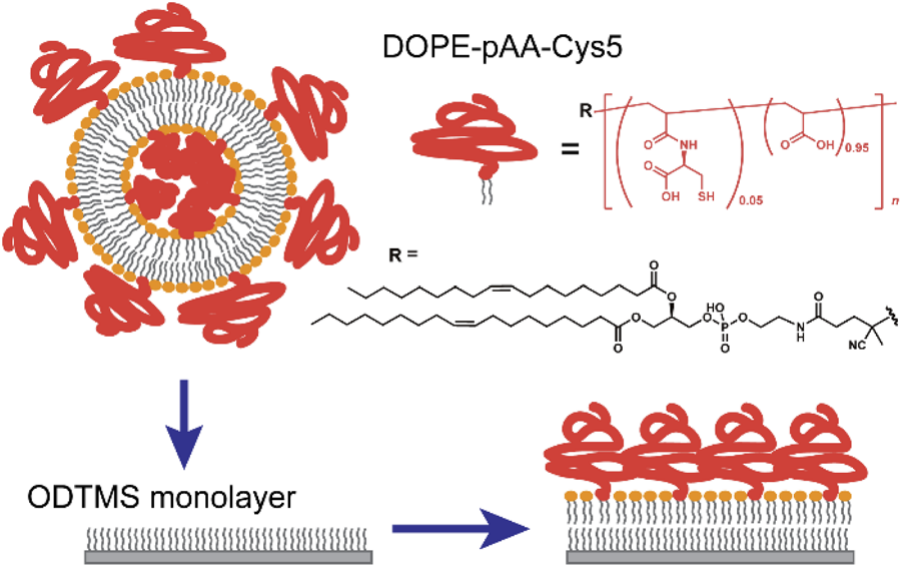
Chemical structure of DOPE-pAA-Cys5 and schematic illustration of the experimental procedure whereby a lipid monolayer is formed from a suspension of small unilamellar vesicles (SUV) and an *N*-octadecyltrimethoxysilane (ODTMS) monolayer on silicon wafers.

In this study, we functionalized the surface of planar lipid membranes (*i.e.*, supported membranes)^22,23^ with poly(acrylic acid) brushes bearing cysteine side chain functional groups (pAA-Cys) by incorporating the lipids covalently coupled with pAA-Cys (*i.e.*, DOPE-pAA-Cys5) into the matrix lipids (Fig. 1). In these materials, pAA-Cys5 moieties interact with divalent Cd^2+^ ions similar to naturally occurring proteins^24^ because pAA-Cys has both –SH and –COOH groups. We investigated how pAA-Cys brushes adapt their structure and mechanical landscape near the interface following the addition of Cd^2+^ ions. We used lipids and lipopolymers with identical hydrocarbon chains to prevent phase separation.^25^ The lateral average distance between lipopolymer molecules 〈*d*〉 can be precisely controlled by taking advantage of the self-assembling nature of lipids and lipopolymers and tuning the molar fraction of lipopolymers *χ*_lipo_, such that

1 〈*d*〉 = (*A*_lipid_/*χ*_lipo_)^0.5^,

where *A*_lipid_ is the cross-sectional area of one lipid molecule (*A*_lipid_ ≈ 0.6 nm^2^).^26,27^ The topographies and 3D frequency shifts in the vicinity of the brush/electrolyte interface in the presence and absence of Cd^2+^ ions were measured using two-dimensional FM-AFM (2D-FM-AFM) and 3D-SFM to monitor the structural and mechanical responses of pAA-Cys brushes to Cd^2+^ ions. Moreover, we examined the ion-specificity of pAA-Cys–Cd^2+^ interactions based on systematic comparisons of the 2D and 3D maps in the presence of Ca^2+^ ions.

## Materials and methods

### Chemicals and reagents

Sodium chloride (NaCl), cadmium chloride (CdCl_2_), toluene, and *n*-butylamine were purchased from Nacalai Tesque (Kyoto, Japan). Calcium chloride (CaCl_2_) was purchased from Wako Pure Chemical Industries, Ltd. (Osaka, Japan). Tris(hydroxymethyl)aminomethane, *S*-trityl-l-cysteine and 2-(dodecylthiocarbonothioylthio)-2-methylpropionic acid (DDMAT) were purchased from Sigma-Aldrich (St. Louis, MO, USA). 1,2-Dioleoyl-*sn-glycero*-3-phosphocholine (DOPC) and 1,2-dioleoyl-*sn-glycero*-3-phosphoethanolamine (DOPE) were purchased from Avanti Polar Lipids (Alabaster, AL, USA). Texas Red™ 1,2-dihexadecanoyl-*sn-glycero*-3-phosphoethanolamine triethylammonium salt (Texas Red™ DHPE) was purchased from Invitrogen (Carlsbad, CA, USA). *N*-Octadecyltrimethoxysilane (ODTMS) was purchased from Fluorochem Ltd. (Derbyshire, UK). Potassium hydroxide (KOH), sodium hydroxide (NaOH), acryloyl chloride, acrylic acid (AA), dimethyl sulfoxide (DMSO), acetone, hexane, trifluoroacetic acid (TFA), diethyl ether, 4,4′-azobis(4-cyanovaleric acid) (ACVA), *N*-hydroxysuccinimide (NHS), *N*,*N*-dimethylformamide (DMF), 4 Å molecular sieves, dichloromethane (DCM), and triethylamine (Et_3_N) were obtained from FUJIFILM Wako Pure Chemical Co. (Osaka, Japan). Ethanol was purchased from Japan Alcohol Trading Co., Ltd. (Tokyo, Japan), and Si wafers were obtained from SUMCO (Tokyo, Japan). Water from a Millipore Integral system (Merck, Darmstadt, Germany) was used throughout this study.

### Synthetic methods

^1^H-NMR spectra were recorded at 400 MHz with a JNM-ECS400 NMR spectrometer (JEOL, Tokyo, Japan). For all NMR measurements, chemical shifts were referenced to the solvent values (*δ* = 2.49 or 7.26 ppm for DMSO-*d*_6_ or CDCl_3_, respectively). Silica gel column chromatography was performed using a Biotage Isolera One instrument (Biotage AB, Uppsala, Sweden) equipped with a SNAP Ultra Column cartridge. Gel permeation chromatography (GPC) measurements of DOPE-pAA-Cys5 was carried out using a high performance liquid chromatography (HPLC) system (CBM-20A/LC-20AD/SIL-10AXL/DGU-20 A3R/CTO-20AC, Shimadzu, Kyoto, Japan) equipped with an SB-804 HQ column (Shodex, Tokyo, Japan) and a refractive index (RI) detector (RID-20A, Shimadzu, Kyoto, Japan), using Tris–HCl buffer (10 mM) containing 100 mM NaCl as an eluent at a flow rate of 0.7 mL min^−1^ at 25 °C. ReadyCal-Kit polyethylene glycol (PEG) (PSS GmbH, Mainz, Germany) was used as the calibration standard. *S*-Trityl-l-cysteine acrylamide (*S*-Tri-Cys-AAm) was prepared according to a previous report.^28^

### Synthesis of ACVA-DOPE

ACVA-DOPE (Fig. S1†) was prepared according to a previous report.^29^ ACVA-NHS (250 mg, 0.53 mmol), DOPE (939 mg, 1.3 mmol), and Et_3_N (175 μL, 1.3 mmol) were dissolved in 50 mL of DCM dried with 4 Å molecular sieves. Volatile chemicals were evaporated after stirring the solution for 3 d at room temperature. Hexane (10 mL) was added to the residue, and the mixture was allowed to stir for 2 d, followed by filtration with a 0.45 μm poly(tetrafluoroethylene) (PTFE) filter. After evaporation of the solvent, ACVA-DOPE was obtained as a colorless oil.

### Synthesis of the DOPE-pAA-Cys5 lipopolymer

DOPE-pAA-Cys5 was synthesized *via* copolymerization of *S*-Tri-Cys-AAm and AA, using ACVA-DOPE as an initiator and DDMAT as a chain transfer agent, followed by deprotection of the trityl group with TFA. Briefly, *S*-tri-Cys-AAm (0.05 mmol), AA (0.95 mmol), ACVA-DOPE (0.01 mmol), and DDMAT (0.01 mmol) were dissolved in 1 mL of ethanol dried with 4 Å molecular sieves. The solution was purged with nitrogen gas for 1 h, sealed, and heated in an oil bath at 70 °C overnight. After cooling to room temperature, the solution was poured into diethyl ether (10 mL) with stirring. The resulting precipitate was collected by centrifugation (3500 rpm, 5 min). After decanting the supernatant, TFA (1 mL) was added, and the mixture was stirred for 1 h at room temperature. The solution was poured into diethyl ether (10 mL). The resulting precipitate was washed with diethyl ether (2 × 10 mL) and dried under vacuum at room temperature. Successful polymerization and deprotection were confirmed by ^1^H-NMR.

### Functionalization of solid substrates with the ODTMS monolayer

Silicon wafers were cut into 10 × 10 mm squares and immersed in a 3 : 1 mixture of H_2_SO_4_/H_2_O_2_ for 15 min to obtain a hydrophilic surface. After thorough rinsing with water, the samples were dried under N_2_ gas and stored in a vacuum chamber at 70 °C. Then, the wafers were immersed in a mixture containing 80 mL toluene, 4.2 mL ODTMS, and 0.4 mL *n*-butylamine, sonicated for 60 min at 10 °C, and let stand for another 30 min. To remove residual chemicals, the samples were rinsed, then sonicated for 2 min in toluene and stored in a vacuum chamber at 70 °C. The quality of the ODTMS coating was confirmed after each preparation based on the water contact angle, *θ* > 90°.^30^

### Preparation of the supported membrane

Mixtures of DOPC/DOPE-pAA-Cys5 = 95 : 5 mol% or DOPC/DOPE-pAA-Cys5/Texas-Red-DHPE = 94 : 5 : 1 mol% were suspended in isopropanol. Lipid dry films were prepared following the gentle evaporation of isopropanol by a flow of N_2_ gas in a glass vial and storage in a vacuum chamber at room temperature overnight. The films were resuspended at a final concentration of 1 mg mL^−1^ in 100 mM NaCl buffered with 10 mM Tris–HCl (pH = 7.4). The products were then sonicated for 30 min at ∼1 W using a tip sonicator (XL2000-600, Misonix, Newtown, CT, USA) and centrifuged (Sorvall Legend Micro 21R, Thermo Scientific, Waltham, MA, USA) to obtain a suspension of small unilamellar vesicles (SUV). The SUV suspensions were then incubated onto the ODTMS-functionalized solid substrates overnight (Fig. 1). After thorough pipetting to remove excessive SUVs, the solution was replaced with fresh 10 mM Tris–HCl buffer solutions (pH = 7.4) containing designated metal ions (*e.g.*, 100 mM NaCl, 100 mM NaCl + 1 mM CaCl_2_, or 100 mM NaCl + 1 mM CdCl_2_) by exchanging the solutions at least 10 times of the initial volume using a pipette. The lateral average distance between the lipopolymer molecules, 〈*d*〉 = 3.6 nm, was calculated from eqn (1), using the cross-sectional area of the single phospholipid membrane *A*_lipid_ ≈ 0.6 nm^2^ and the molar fraction of lipopolymers *χ*_lipo_ = 0.05.

### Optical microscopy

Fluorescence microscopy images were captured with a Zeiss Axiovert microscope (Carl Zeiss, Oberkochen Germany) equipped with a digital CMOS camera (ORCA-Flash4.0, Hamamatsu Photonics, Shizuoka, Japan) and an LED illumination system (X-Cite 120LED, Excelitas technologies, Waltham, MA, USA).

### AFM measurements

AFM measurements were performed using a custom-built AFM instrument with an ultralow-noise cantilever deflection sensor and a highly stable photothermal cantilever excitation system.^31–33^ The AFM was controlled with a commercially available controller (ARC2, Oxford Instruments, Oxfordshire, UK) using a modified software. A commercially available phase-locked loop circuit (Nanonis OC4, SPECS, Berlin, Germany) was used to oscillate the cantilever at its resonance frequency in liquid *f*_0_ with a constant amplitude and to detect the frequency shift Δ*f* induced by the force variation. The cantilevers (160AC, OPUS, Sofia, Bulgaria) with a nominal spring constant *k* = 26 N m^−1^ were used. The *f*_0_ and the quality factor *Q* were determined for each measurement; *f*_0_ = 120.2 kHz and *Q* = 7.8 in 100 mM NaCl, *f*_0_ = 137.9 kHz and *Q* = 8.0 in 100 mM NaCl + 1 mM CaCl_2_, and *f*_0_ = 139.0 kHz and *Q* = 8.9 in 100 mM NaCl + 1 mM CdCl_2_. The cantilever tips were coated with an Si thin film using a sputter coater (KST-CSPS-KF1, K's Tech, Ibaraki, Japan) to obtain an apex diameter of approximately 20 nm.^34^

For the 2D-FM-AFM measurements, a square 500 × 500 nm area was scanned at a scan rate of 1 Hz (512 × 512 pixels), with the setpoint Δ*f*_sp_ = 488 Hz.

For the 3D-SFM measurements, the cantilever tip scanned vertically following a rapid sinusoidal curve, while slowly scanning in the lateral direction. During this tip scan, the Δ*f* induced by the force variation was recorded to produce a 3D Δ*f* image; the tip-sample distance was regulated continuously such that the average Δ*f* was equal to a setpoint value Δ*f*_sp_. This allowed us to simultaneously obtain a 3D Δ*f* image and a 2D height image. Sections of 100 × 100 nm in area with a height of 6 nm (for NaCl and NaCl + CaCl_2_ solutions) or 8 nm (for NaCl + CdCl_2_ solutions) were scanned (128 × 128 × 256 pixels) with a constant setpoint to compare the forces measured in all the buffer solutions. In this study, we carefully optimized the setpoint at 3.9 kHz, at which we could gain the highest contrast.

### Data analysis

AFM data were processed with the open-source software, Gwyddion,^35^ to analyze the topography images and a self-written program in LabVIEW (National Instruments, Austin, TX, USA) to analyze the 3D-SFM images.

The autocorrelation analysis of the topography images was conducted using a self-written algorithm in MATLAB 2021b (Natick, MA, USA). For a given image Δ*h*(*x*,*y*) consisting of (*m* × *n*) pixels, the autocorrelation *G* can be calculated as follows:2
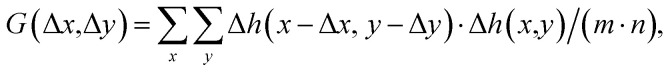
where Δ*x* and Δ*y* represent the lag from the corresponding *x* and *y* positions, respectively. However, this definition is computationally laborious. Therefore, the autocorrelation of an image was calculated using the Wiener–Khinchin theorem *via* fast Fourier transforms, which is defined as

3 *G* = *F*^−1^{|*F*(Δ*h*(*x*,*y*))|^2^}/(*m*·*n*)

where *F* and *F*^−1^ are the fast Fourier transform and inverse fast Fourier transform, respectively.

The vertical Δ*f* curves plotted as a function of the height were converted to force curves according to Sader's model,^36^ where the interaction force between the tip and sample *F* is expressed as shown in eqn (4),4
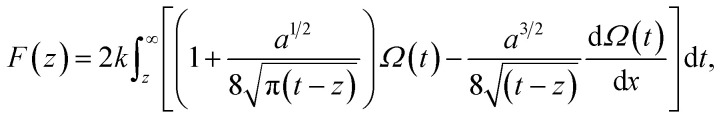
where *Ω*(*t*) = Δ*ω*(*z*)/*ω*_0_, with *ω*_0_ = 2π*f*_0_ and Δ*ω* = 2πΔ*f*, and *a* is the amplitude of the oscillation. The processed data were further analyzed in IGOR Pro (WaveMetrics, Portland, OR, USA).

## Results and discussion

### Synthesis and characterization of DOPE-pAA-Cys5

First, DOPE-pAA-Cys5 was synthesized *via* reversible addition–fragmentation chain transfer (RAFT) copolymerization using ACVA-DOPE as a radical initiator with a DOPE moiety. Fig. S1† shows the ^1^H-NMR spectra of ACVA-DOPE and DOPE-pAA-Cys5. Peaks corresponding to ACVA-DOPE were also observed in the NMR spectrum of DOPE-pAA-Cys5. Fig. S2† shows the GPC trace of DOPE-pAA-Cys5, which gave a unimodal peak with *M*_w_ = 7.1 × 10^3^ (PEG standard) and *M*_w_/*M*_n_ = 1.9. These results indicate that the polymer was successfully synthesized with DOPE at the end.

### Formation of supported lipid membranes doped with the lipopolymer

**Fig. 2:**
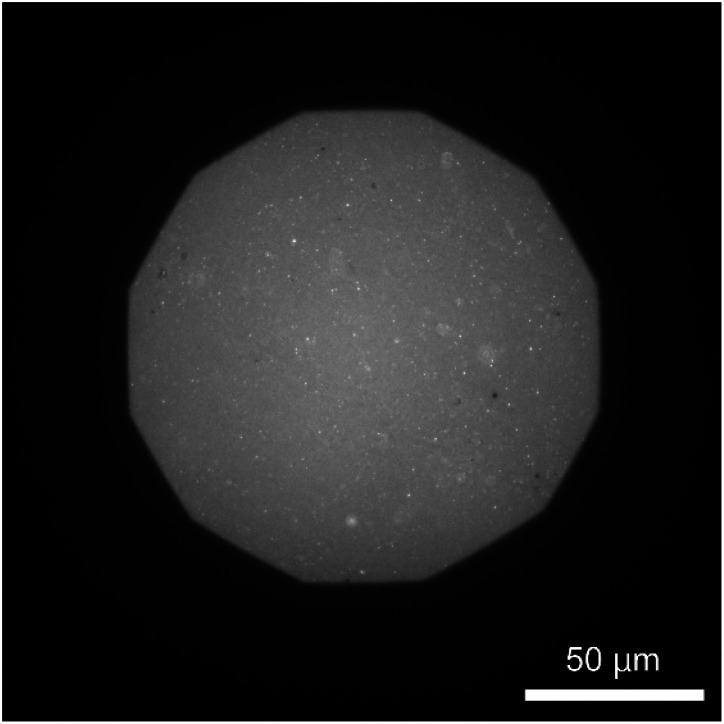
Fluorescence image of a supported membrane (DOPC/DOPE-pAA-Cys5/Texas Red DHPE = 94 : 5 : 1 mol%) on a glass cover slip coated with an ODTMS monolayer, verifying the formation of a uniform lipid monolayer incorporating DOPE-pAA-Cys5. Scale bar: 50 μm.

The formation of a DOPC monolayer incorporating 5 mol% DOPE-pAA-Cys5 was monitored by doping 1 mol% Texas Red-DHPE (refer to the Materials and methods section). The acquired image (Fig. 2) shows a uniform fluorescence signal, indicating that the lipids and lipopolymers formed a homogeneous monolayer on the hydrophobic ODTMS surface with no sign of phase separation. It is noted that the circular bright spots in the image could be SUVs remaining on the supported membrane after rinsing.

### Cd^2+^ ions induce roughening of the pAA-Cys5 polymer brush

**Fig. 3:**
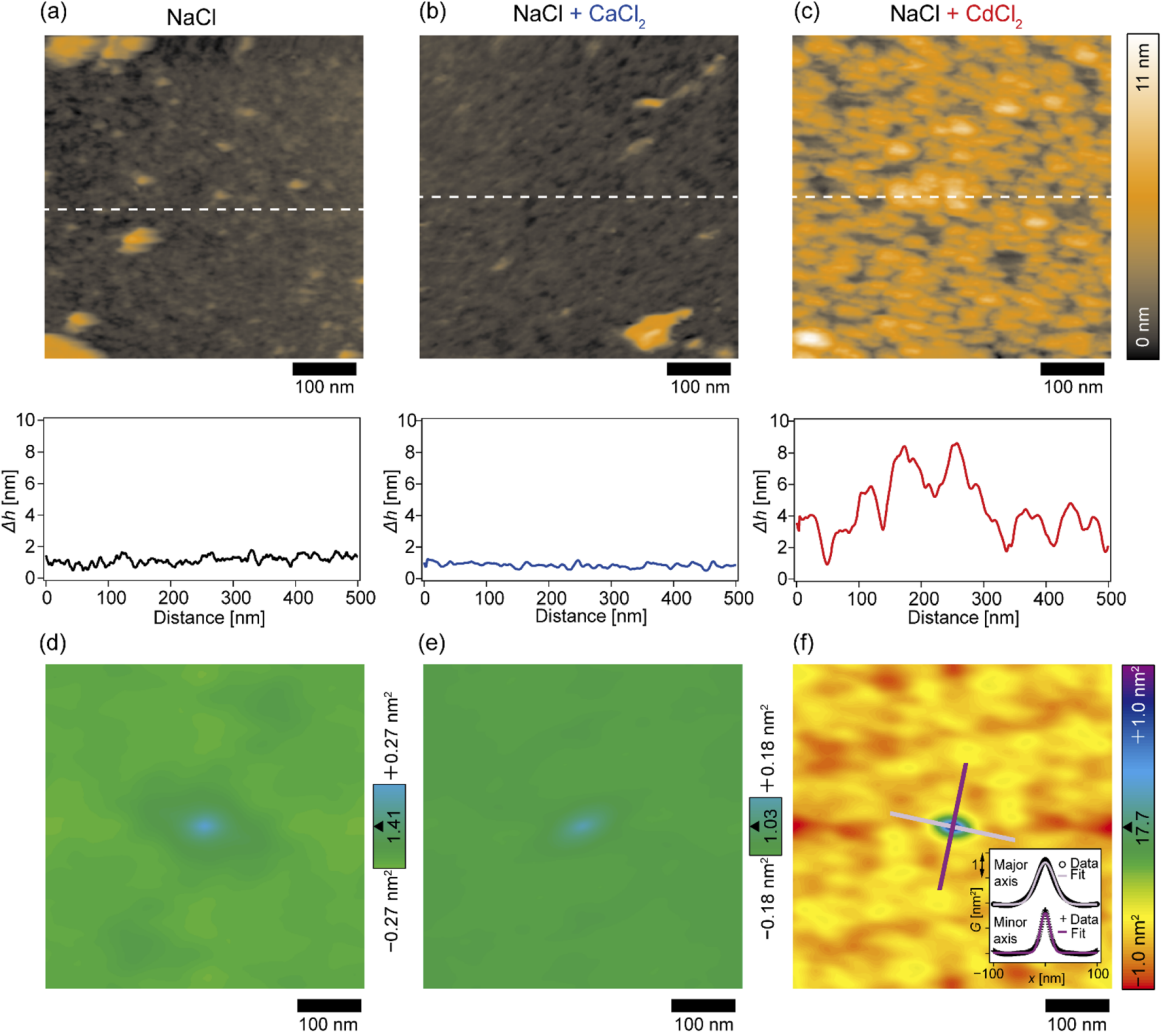
2D-FM-AFM topography measurements of pAA-Cys5 polymer brushes. (a–c, top) 2D images of polymer brushes in (a) 100 mM NaCl, (b) 100 mM NaCl + 1 mM CaCl_2_, and (c) 100 mM NaCl + 1 mM CdCl_2_, and (bottom) line profiles obtained at the dashed lines in 2D images. Scale bars: 100 nm. (d–f) Autocorrelation maps of the topography images shown in (a–c); (d) 100 mM NaCl, (e) 100 mM NaCl + 1 mM CaCl_2_, and (f) 100 mM NaCl + 1 mM CdCl_2._ Scale bars: 100 nm. (f, inset) The line profiles along the major and minor axes of the central peak and their best Gaussian fits. The profiles are shifted vertically for the clarity.

The topographic profiles of the DOPC/DOPE-pAA-Cys5 monolayer surface in the absence or presence of divalent cations were characterized using 2D-FM-AFM. The minimum positional height Δ*h* in the field of view is defined as Δ*h* = 0 nm. In the absence of divalent cations, the surface of the pAA-Cys5 brush had a smooth topographic profile (Fig. 3a, top). The line profile corresponding to the dashed line indicates that the height fluctuation was well below ±1 nm (Fig. 3a, bottom). The root mean square (RMS) roughness calculated from the 100 × 100 nm area at the center was RMS_0_ = 0.23 nm. When 1 mM CaCl_2_ was added to the solution, the brush surface remained smooth, with a RMS_Ca_ = 0.15 nm (Fig. 3b). Remarkably, the topographic profile of the brush surface was distinct in the presence of 1 mM CdCl_2_, exhibiting dense hemi-ellipsoidal protrusions (Fig. 3c, top). The calculated RMS_Cd_ = 1.47 nm was significantly larger than those obtained under the other studied conditions.

To examine whether pAA-Cys5 brushes adopt specific structural features in the absence or presence of divalent cations, we calculated the autocorrelations of the topographical images (Fig. 3d–f) *via* fast Fourier transformation within the framework of the Wiener–Khinchin theorem (refer to the Materials and methods section). As presented in Fig. 3d, the autocorrelation map reveals no characteristic features in the absence of divalent cations. Similarly, the autocorrelation function remained featureless when 1 mM CaCl_2_ was added to the solution (Fig. 3e). The featureless autocorrelation maps in Fig. 3d and e are consistent with the uniform topographic profiles in Fig. 3a and b. In contrast, when 1 mM CdCl_2_ was added to the solution, the autocorrelation map (Fig. 3f) showed a distinct pattern. First, we found an intense peak in the center of the autocorrelation map corresponding to the characteristic sizes of protrusions. From the full width at half maximum (FWHM) of the intensity profile along the major and minor axes (Fig. 3f, inset), the characteristic lengths were determined to be 41 nm and 22 nm, respectively. It should be noted that these values are much larger than the lateral average distance between lipopolymer molecules 〈*d*〉 = 3.6 nm. Even though the object size might be overestimated due to technical artifacts such as the finite size effect of the cantilever tip^37,38^ and a thermal drift during scanning, each protrusion might consist of multiple polymer chains. Secondly, similarly intense local maxima were observed around the central peak in the autocorrelation map, which indicates that these protrusions were densely packed. However, there is no clear pattern in the autocorrelation map, suggesting that these protrusions do not take any distinct pattern in the presence of Cd^2+^. To confirm this point, we performed the fast Fourier transform (FFT) of the topographic profile (Fig. S3†) and found no distinct patterns, suggesting that these protrusions do not form a 2D lattice. This finding seems reasonable as both lipids and lipopolymers are in a fluid Lα phase, in which hydrocarbon chains are disordered and uniformly mixed. It is also notable that the central ellipse showed a slight tilt with respect to the horizontal scanning direction, which might be caused by a thermal drift. However, we were not able to correct the topographic profiles, because there was no reference pattern with clear order.^39,40^ Thus, although the topographic profiles might be distorted due to drift, the autocorrelation analysis suggested that pAA-Cys5 brushes formed uniformly sized protrusions and assembled randomly only in the presence of Cd^2+^ ions.

### Cd^2+^ ions induce an inhomogeneous Δ*f* field near the interface

**Fig. 4:**
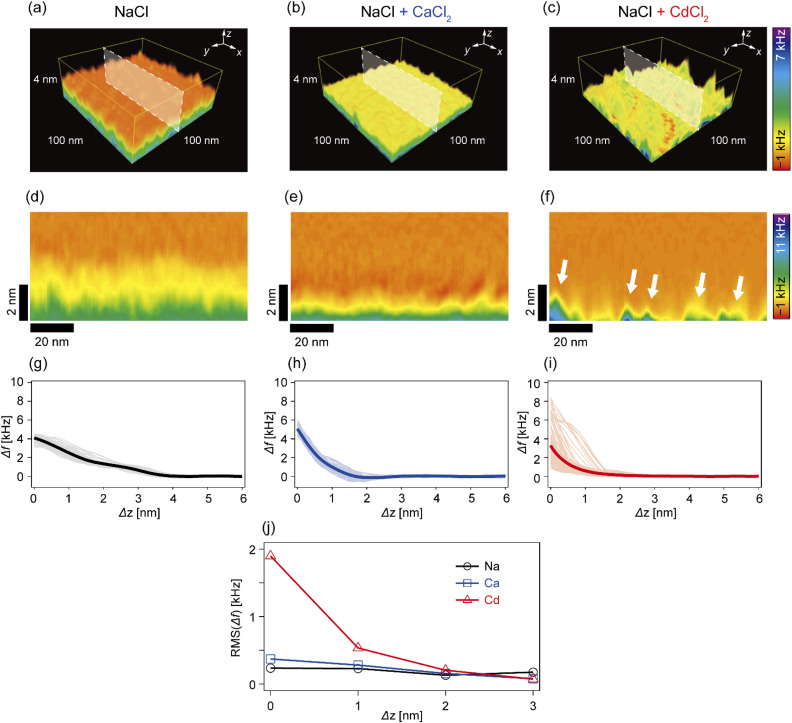
3D-SFM measurements of pAA-Cys5 polymer brushes. (a–c) 3D Δ*f* maps in (a) 100 mM NaCl, (b) 100 mM NaCl + 1 mM CaCl_2_, and (c) 100 mM NaCl + 1 mM CdCl_2_. (d–f) *xz* cross-sections of Δ*f* images in (d) 100 mM NaCl, (e) 100 mM NaCl + 1 mM CaCl_2_, and (f) 100 mM NaCl + 1 mM CdCl_2_, corresponding to the white shaded planes in the 3D Δ*f* maps in (a–c). Scale bars: 20 nm in the horizontal direction and 2 nm in the vertical direction. (g–i) Individual Δ*f*–Δ*z* curves (thin lines) extracted from (d–f), and their averages (thick lines); (g) 100 mM NaCl, (h) 100 mM NaCl + 1 mM CaCl_2_, and (i) 100 mM NaCl + 1 mM CdCl_2_. (j) Root mean squared “roughness” of Δ*f* as a function of Δ*z*.

In the next step, we investigated how Cd^2+^ and Ca^2+^ ions modulated the nanoscopic mechanical landscape in the vicinity of pAA-Cys5 brush surfaces. Fig. 4a–c show the 3D Δ*f* maps over a 100 × 100 nm × 4 nm area. The bottom planes of each 3D map (where Δ*z* = 0 nm) coincide with the topographical surfaces determined with a setpoint value of Δ*f*_sp_ (refer to the Materials and methods section), which can vary at each *xy* position. A transparency filter was applied to the color map to visualize the high Δ*f* regions (Fig. S4†). The pAA-Cys5 brushes in NaCl solution showed uniformly low Δ*f* values near the surface (Fig. 4a). Notably, Δ*f* gradually converged to the level in the bulk. The 3D Δ*f* map in the presence of an additional 1 mM CaCl_2_ (Fig. 4b) also shows a uniform profile near the surface. However, in this case, the transition to the bulk occurred more sharply at a shorter distance, which suggested uniform compaction of pAA-Cys5 brushes. In the presence of an additional 1 mM CdCl_2_ (Fig. 4c), the 3D Δ*f* map is distinct. The 3D maps reveal heterogeneous domains, similar to those observed in the 2D topographical map (Fig. 3c).

To gain further insight into the transition from the brush to the bulk electrolyte, we extracted the *xz* cross-sections from the 3D Δ*f* maps. As shown in Fig. 4d, the Δ*f* value of pAA-Cys5 in NaCl decayed to that of the bulk level over a depth of 4 nm. This relatively broad transition from the polymer to the bulk electrolyte can be attributed to weak forces originating from the conformational fluctuations of hydrated pAA-Cys5 brushes. In the electrolyte containing additional Ca^2+^ (Fig. 4e), the width of the transition zone was approximately 2 nm, which was narrower than in the NaCl buffer. This result suggests that pAA-Cys5 brushes became compact in the presence of Ca^2+^, similar to other negatively charged polymers.^41–43^ Remarkably, the *xz* cross-section of pAA-Cys5 in the presence of Cd^2+^ (Fig. 4f) was generally heterogeneous in the *x*-direction, which is different from the other conditions. Some regions had Δ*f* values comparable to those of the bulk phase and were attributed to gaps/defects. In fact, the lateral size of “triangular” features with high Δ*f* values near Δ*z* = 0 nm (indicated by white arrows) was similar to that of the hemi-ellipsoidal protrusions observed in the 2D topography map (Fig. 3c). Fig. 4g–i present the frequency shift curves (Δ*f*–Δ*z*), which highlight the differences in the shapes and widths of the “polymer-to-electrolyte transitions”. The thick solid lines represent the mean of 128 curves collected from the *xz* cross-sections shown in Fig. 4d–f. In the buffer containing only NaCl (Fig. 4g), the onset of Δ*f* increase was observed at Δ*z* ≈ 4 nm. The individual Δ*f*–Δ*z* profiles overlap with one another, indicating that the mechanical properties near the brush/electrolyte interface are uniform over the surface. In the buffer containing additional Ca^2+^ ions (Fig. 4h), Δ*f* begins increasing at Δ*z* ≈ 2 nm. When 1 mM CdCl_2_ was added to the buffer (Fig. 4i), the Δ*f*–Δ*z* profiles become significantly more heterogeneous. Most Δ*f*–Δ*z* profiles show an onset of increase at Δ*z* ≈ 2 nm, while others show an increase starting at Δ*z* ≤ 1 nm. The RMS “roughness” of Δ*f* was plotted as a function of Δ*z* to obtain another measure of the heterogeneity of mechanical properties near the brush/electrolyte interface (Fig. 4j). The presence of Cd^2+^ ions led to an inhomogeneous Δ*f* profile in the vicinity of Δ*z* = 0 nm, although this heterogeneous Δ*f* distribution converges to that of the other conditions already at Δ*z* ≥ 2 nm.

### Ion-specific modulation of the mechanical properties of pAA-Cys5 brushes

**Fig. 5:**
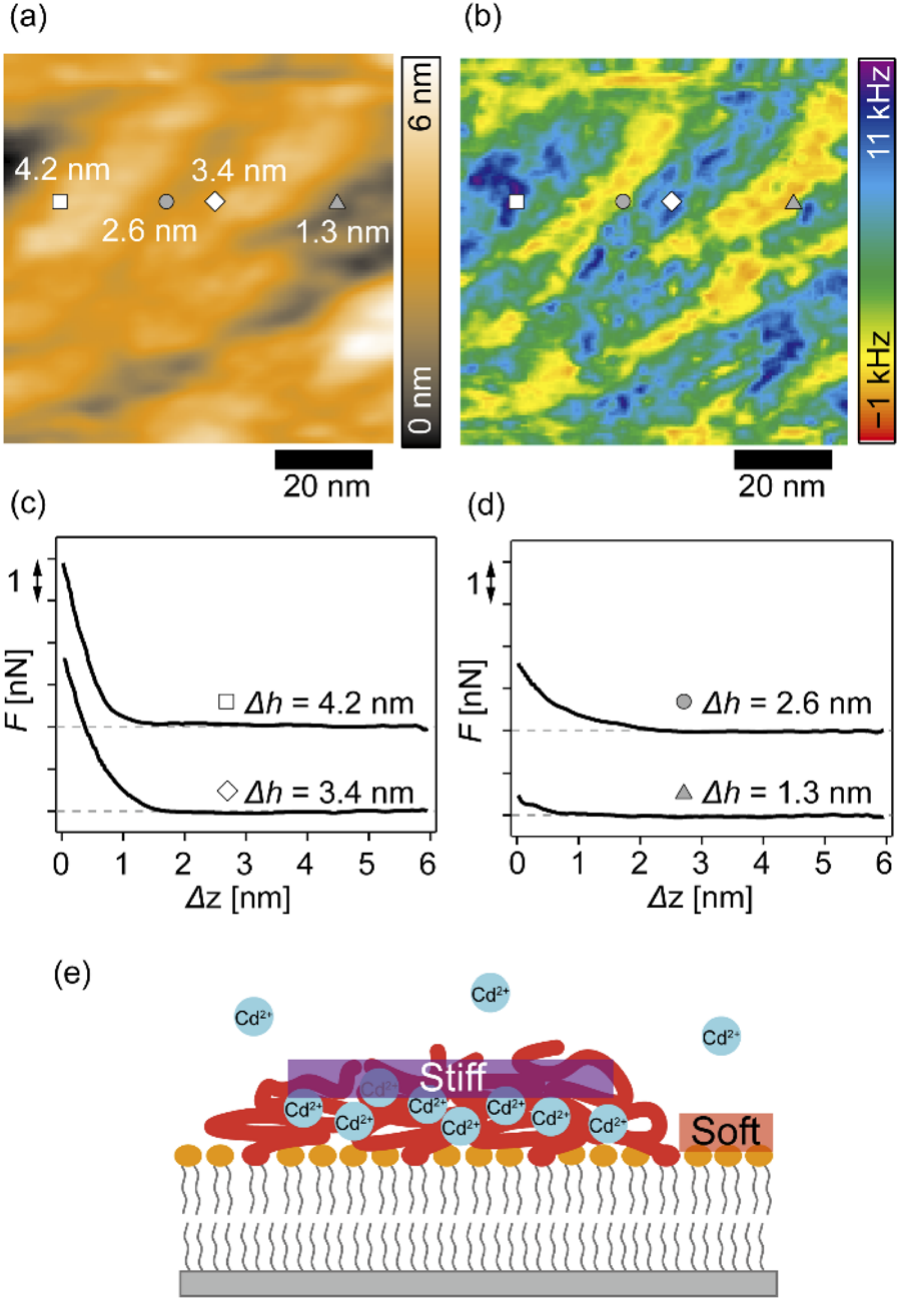
Correlations between the surface topography and mechanical heterogeneity of pAA-Cys5 polymer brushes in 100 mM NaCl + 1 mM CdCl_2_ based on 3D-SFM mapping. (a) Surface topography. A white square and a white diamond indicate the positions of representative topographically high points (Δ*h* = 4.2 and 3.4 nm, respectively), and a grey circle and a grey triangle indicate the positions of representative topographically low points (Δ*h* = 2.6 and 1.3 nm, respectively). (b) 2D map of Δ*f* at the Δ*z* = 0 plane. Scale bars: 20 nm. (c and d) Force curves measured at (c) the protrusions and (d) the smooth regions; baselines are offset for clarity. (e) A schematic presentation of pAA-Cys5 brushes on a phospholipid monolayer and lateral stiffness distribution in the presence of Cd^2+^ ions.

We further analyzed the 3D-SFM data to examine whether the topographical heterogeneity characterized by hemi-ellipsoidal protrusions was correlated with the mechanical heterogeneity of pAA-Cys5 brushes in the presence of Cd^2+^ ions. Fig. 5a shows the Δ*h* profile of the pAA-Cys5 brush surface in the presence of 1 mM CdCl_2_ scanned over an area of 100 × 100 nm. The corresponding Δ*f* profile at Δ*z* = 0 nm measured by 3D-SFM is presented in Fig. 5b. We selected four representative points: two near the top of the protrusions (Δ*h* = 4.2 and 3.4 nm, indicated by a white square and a white diamond, respectively) and two in the lower, smoother regions (Δ*h* = 2.6 and 1.3 nm, indicated by a grey circle and a grey triangle, respectively). Using eqn (4), the Δ*f* obtained from 3D-SFM can be converted into a force *F* to obtain force curves. Fig. 5c and d show the force curves from the protrusions and smooth regions, respectively. The increase in *F* during the approach is notably sharper in the protrusions (Fig. 5c) relative to that in the smooth regions (Fig. 5d), which suggests that the protrusions are stiffer than the smooth regions. In general, such a force–distance relationship can be used to calculate Young's modulus *E* of the films using a modified Hertz model^44,45^ as expressed in eqn (5),5
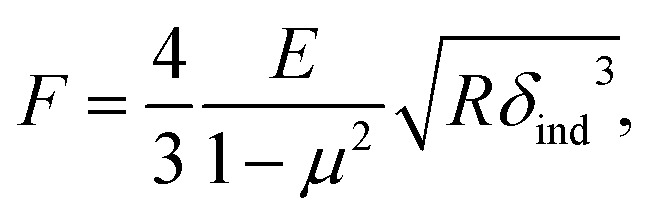
where *δ*_ind_ is the indentation depth, *μ* is Poisson's ratio, and *R* is the tip radius. However, it is well established that AFM indentation data for a thin film on a stiff substrate cannot be treated with a conventional Hertz model because the film deformation is limited under a high load.^46,47^ If the film is softer than the underlying solid substrate, one can analytically calculate Young's modulus of very thin, soft films using a transition function that linearly connects the influence of two elastic layers, *i.e.*, the film and the substrate^48,49^ as shown in eqn (6),6
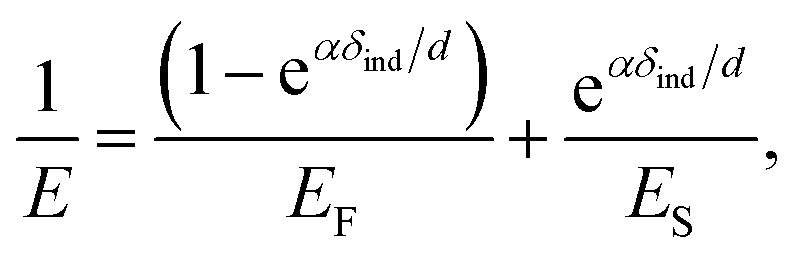
where *E* is the combined Young's modulus, *E*_F_ and *E*_S_ are Young's moduli of the film and substrate, respectively, and *d* is the film thickness. In eqn (6), *α* is an empirical parameter characterizing the sharpness of the transition. Previously, we applied this model to calculate Young's moduli of dendronized oligoethylene glycol monolayers and determined how the number and length of branches affected the film elasticity.^50^ However, this approach was not used in the present study because the experimental system consists of five distinct layers: the (i) Si wafer, (ii) ODTMS monolayer, (iii) phospholipid monolayer, (iv) pAA-Cys5 brush layer, and (v) electrolyte. Within the framework of this model, the sharpness of the transition at each interface is approximated by using an empirical parameter. Moreover, the precise determination of each layer's thickness is a prerequisite for calculating Young's modulus with the transition function,^48,49^ which is non-trivial in this multilayered system. Furthermore, it is possible that high-speed indentation at an oscillation frequency of 130 kHz may result in an overestimation of Young's modulus because the viscoelastic response of the soft polymer brush interface may not be accurately captured.^51^

Additionally, the lateral shift between the Δ*h* map (Fig. 5a) and the Δ*f* map at Δ*z* = 0 nm (Fig. 5b) appeared because during the 3D-SFM measurements, the feedback gain was optimized for the vertical sinusoidal oscillation of the cantilever. This enabled the sensitive detection of Δ*f* without scratching the surface; however, it also caused a slight delay in the adjustment of the average vertical position of the cantilever. Although a quantitative *E* map could not be obtained, a direct comparison of Δ*h* and Δ*f* maps enables us to connect the surface topography and mechanical properties on the nanoscale. Currently we interpret that the “stiffer protrusions” correspond to collapsed pAA-Cys5 brushes and the “softer, smoother regions” to the underlying phospholipid monolayer (Fig. 5e).

Fig. S5† shows the overlayed force curves extracted from Fig. 4g–i. Similar to the corresponding Δ*f* curves (Fig. 4g), the force curves in NaCl buffer overlap with one another, showing a slow increase to *F* = 3.5 nN at the interface with the onset of *F* increase at Δ*h* = 4 nm (Fig. S5a†). This is reasonable because the interface between hydrated polyelectrolyte brushes and the electrolyte is diffusive.^52,53^ In the presence of an additional 1 mM CaCl_2_, the onset of *F* increase was observed at a shallower position (Δ*h* = 2 nm), and *F* increased to *F* = 2 nN at the interface (Fig. S5b†). A minor deviation in the force curves indicated that the pAA-Cys5 brushes were uniformly compacted and therefore stiffened following the binding of Ca^2+^ ions. Interestingly, the force curves in the presence of Cd^2+^ ions showed a much more pronounced deviation (Fig. S5c†). Both the onset of *F* increase and the *F* value at Δ*h* = 0 nm exhibited wide distributions, implying that pAA-Cys5 brushes formed stiff domains on the surface of the phospholipid monolayer. The formation of collapsed domains in the presence of Cd^2+^ ions suggested that the compaction-induced dehydration of pAA-Cys5 brushes altered the affinity of the solvent (water) to monomers. Further investigations, including *in situ* observations of changes in the viscoelasticity of pAA-Cys5 using 3D-SFM or other techniques (*e.g.*, QCM-D) would provide further insights into the ion-specific, dynamic modulation of polyelectrolyte brushes.

## Conclusions

In this study, we investigated the modulation of topography and mechanical properties of supported lipid monolayers incorporating lipids functionalized with linear poly(acrylic acid) chains with cysteine side chains (DOPE-pAA-Cys5) induced by different metal ions. We found that the topographical profile of pAA-Cys5 brushes obtained by 2D-FM-AFM in the electrolyte containing no divalent cation (100 mM NaCl, 10 mM Tris–HCl, pH = 7.4) was very homogeneous, whose RMS_0_ = 0.23 nm (Fig. 3a). In fact, autocorrelation analysis of Δ*h* showed no clear feature (Fig. 3d). The mechanical landscape, characterized by the 3D Δ*f* map (Fig. 4a) and its *xz* cross-section (Fig. 4d), was also highly uniform, suggesting that the hydrated pAA-Cys5 brushes are stretched into the bulk electrolyte. The slow increase in the Δ*f*–Δ*z* curves (Fig. 4g) and a very low RMS(Δ*f*) = 0.24 kHz at Δ*z* = 0 nm (Fig. 4j) implied a smooth transition from the polymer to the bulk electrolyte.

The addition of 1 mM CaCl_2_ caused a distinct change in both topographical and mechanical properties of the brush/electrolyte interface. In the presence of Ca^2+^ ions, pAA-Cys5 brushes became smoother, which could be characterized by the very small roughness, RMS_Ca_ = 0.15 nm (Fig. 3b) with no characteristic feature (Fig. 3e). The 3D Δ*f* map and its *xz* cross-section (Fig. 4b and e) were smoother, and the increase in Δ*f* was much sharper (Fig. 4h) than the corresponding values obtained in the absence of Ca^2+^ ions, RMS(Δ*f*) = 0.37 kHz at Δ*z* = 0 nm (Fig. 4j), indicating that pAA-Cys5 brushes are uniformly compacted by the presence of Ca^2+^ ions. The most remarkable difference was observed when 1 mM CdCl_2_ was added to the electrolyte. The surface topography became very rough (RMS_Cd_ = 1.47 nm, Fig. 3c), displaying hemi-ellipsoidal features. The autocorrelation analysis suggested that these protrusions had a similar size and assembled randomly (Fig. 3f). Accordingly, the 3D Δ*f* map became highly heterogeneous (Fig. 4c), and the *xz* cross-section (Fig. 4f) clearly indicates the coexistence of stiff protrusions and defects. The heterogeneity of the mechanical landscape was clearly visible from a broad distribution of the Δ*f*–Δ*z* curves, resulting in RMS(Δ*f*) = 1.90 kHz at Δ*z* = 0 nm (Fig. 4j). These data suggest that pAA-Cys5 brushes “collapsed” in the presence of Cd^2+^ ions, where water might not be a good solvent for pAA-Cys5.

3D-SFM also provides with a unique possibility to correlate nanoscale heterogeneities in the surface topography (Fig. 5a) and the mechanical landscape (Fig. 5b) observed in the presence of Cd^2+^ ions. The force curves extracted from 2 × “higher” positions (Fig. 5c) and 2 × “lower” positions (Fig. 5d) showed distinctly different increases. Although the quantification of mechanical parameters, such as Young's modulus, from the force curves was not possible with our multilayered lipid/polymer systems, a sharper increase in Δ*f* shown in Fig. 5c suggests that the higher regions (protrusions) are stiffer than the lower region showing a markedly slower increase, as schematically shown in Fig. 5e.

It should be noted that the change in chain conformation and mechanical properties of the polymer brush induced by Cd^2+^ ions are reversible, as we recently demonstrated by using quartz-crystal microbalance with dissipation (QCMD).^54^ The compaction and stiffening of the polymer brush due to Cd^2+^ ions observed in the current study coincide well with the decrease in the frequency and the increase in the energy dissipation measured by QCMD. We currently interpret that the Cd^2+^-specific compaction of pAA-Cys5 brushes originates from the coexistence of both –SH and –COOH moieties, which share common features with the naturally occurring proteins that form coordination complexes with heavy metal ions, because the polymers lacking either –SH or –COOH moieties showed no remarkable response to Cd^2+^ ions.^54^ More detailed structural studies on the coordination complex analysis with NMR or the change in vibrational bands using infrared spectroscopy will allow for the molecular-level mechanism understanding of the observed ion-specific response and the rational design of adaptable polymer brushes that are modulated by specific ions.

## Author contributions

M. T. and K. M. designed and directed the research. M. N. synthesized and characterized the lipopolymers. A. Y., T. I., K. M., and R. S. performed the experiments and analysed the data. A. Y., K. M., T. F., M. N., and M. T. wrote the manuscript, and all the authors were involved in the discussion throughout the manuscript preparation.

## Conflicts of interest

There are no conflicts to declare.

## Supplementary Material

NA-004-D2NA00350C-s001

## References

[cit1] Stuart M. A., Huck W. T., Genzer J., Muller M., Ober C., Stamm M., Sukhorukov G. B., Szleifer I., Tsukruk V. V., Urban M., Winnik F., Zauscher S., Luzinov I., Minko S. (2010). Nat. Mater..

[cit2] Rühe J., Ballauff M., Biesalski M., Dziezok P., Gröhn F., Johannsmann D., Houbenov N., Hugenberg N., Konradi R., Minko S., Motornov M., Netz R. R., Schmidt M., Seidel C., Stamm M., Stephan T., Usov D., Zhang H. (2004). Adv. Polym. Sci..

[cit3] Zhou F., Huck W. T. (2006). Phys. Chem. Chem. Phys..

[cit4] Ballauff M., Borisov O. (2006). Curr. Opin. Colloid Interface Sci..

[cit5] Russell T. P. (1996). Phys. B.

[cit6] Takahara A., Higaki Y., Hirai T., Ishige R. (2020). Polymers.

[cit7] Moya S., Azzaroni O., Farhan T., Osborne V. L., Huck W. T. (2005). Angew. Chem., Int. Ed. Engl..

[cit8] Hollingsworth N. R., Wilkanowicz S. I., Larson R. G. (2019). Soft Matter.

[cit9] Spencer N. D. and Tysoe W. T., The Cutting Edge of Tribology, World Scientific Publishing, 2015

[cit10] Sinha S. K. and Briscoe B. J., Polymer Tribology, Imperial College Press, Distributed by World Scientific, London, Singapore, Hackensack, NJ, 2009

[cit11] Higaki Y., Frohlich B., Yamamoto A., Murakami R., Kaneko M., Takahara A., Tanaka M. (2017). J. Phys. Chem. B.

[cit12] Monzel C., Veschgini M., Madsen J., Lewis A. L., Armes S. P., Tanaka M. (2015). Langmuir.

[cit13] Cuellar J. L., Llarena I., Moya S. E., Donath E. (2013). Macromolecules.

[cit14] Farhan T., Azzaroni O., Huck W. T. S. (2005). Soft Matter.

[cit15] Fukuma T. (2010). Sci. Technol. Adv. Mater..

[cit16] Songen H., Reischl B., Miyata K., Bechstein R., Raiteri P., Rohl A. L., Gale J. D., Fukuma T., Kuhnle A. (2018). Phys. Rev. Lett..

[cit17] Fukuma T., Ueda Y., Yoshioka S., Asakawa H. (2010). Phys. Rev. Lett..

[cit18] Yang C. W., Miyazawa K., Fukuma T., Miyata K., Hwang I. S. (2018). Phys. Chem. Chem. Phys..

[cit19] Foster W., Miyazawa K., Fukuma T., Kusumaatmaja H., Voiotatchovsky K. (2020). Nanoscale.

[cit20] Asakawa H., Yoshioka S., Nishimura K.-i., Fukuma T. (2012). ACS Nano.

[cit21] Ikarashi T., Yoshino T., Nakajima N., Miyata K., Miyazawa K., Morais Jaques Y., Foster A. S., Uno M., Takatoh C., Fukuma T. (2020). ACS Appl. Nano Mater..

[cit22] Sackmann E. (1996). Science.

[cit23] Tanaka M., Sackmann E. (2005). Nature.

[cit24] Hamer D. H. (1986). Annu. Rev. Biochem..

[cit25] Purrucker O., Fortig A., Jordan R., Tanaka M. (2004). ChemPhysChem.

[cit26] Kaindl T., Rieger H., Kaschel L. M., Engel U., Schmaus A., Sleeman J., Tanaka M. (2012). PLoS One.

[cit27] Rieger H., Yoshikawa H. Y., Quadt K., Nielsen M. A., Sanchez C. P., Salanti A., Tanaka M., Lanzer M. (2015). Blood.

[cit28] Podasca V. E., Buruiana T., Varganici C. D., Buruiana E. C. (2017). J. Polym. Res..

[cit29] Wang H., Chen Z., Xin L., Cui J., Zhao S., Yan Y. (2015). J. Polym. Sci., Part A: Polym. Chem..

[cit30] Hillebrandt H., Tanaka M. (2001). J. Phys. Chem. B.

[cit31] Fukuma T., Jarvis S. P. (2006). Rev. Sci. Instrum..

[cit32] Fukuma T. (2009). Rev. Sci. Instrum..

[cit33] Fukuma T., Kimura M., Kobayashi K., Matsushige K., Yamada H. (2005). Rev. Sci. Instrum..

[cit34] Akrami S. M. R., Nakayachi H., Watanabe-Nakayama T., Asakawa H., Fukuma T. (2014). Nanotechnology.

[cit35] Nečas D., Klapetek P. (2012). Open Phys..

[cit36] Sader J. E., Jarvis S. P. (2004). Appl. Phys. Lett..

[cit37] Engel A., Schoenenberger C.-A., Müller D. J. (1997). Curr. Opin. Struct. Biol..

[cit38] Tutus M., Rossetti F. F., Schneck E., Fragneto G., Forster F., Richter R., Nawroth T., Tanaka M. (2008). Macromol. Biosci..

[cit39] Jones L., Nellist P. D. (2013). Microsc. Microanal..

[cit40] Wu Y., Fan Z., Fang Y., Liu C. (2021). IEEE Trans. Instrum. Meas..

[cit41] Xu X., Mastropietro D., Ruths M., Tirrell M., Yu J. (2019). Langmuir.

[cit42] Kundagrami A., Muthukumar M. (2008). J. Chem. Phys..

[cit43] Schneck E., Papp-Szabo E., Quinn B. E., Konovalov O. V., Beveridge T. J., Pink D. A., Tanaka M. (2009). J. R. Soc., Interface.

[cit44] Hertz H. (1882). für die Reine und. *Angew. Math.*.

[cit45] Butt H.-J., Cappella B., Kappl M. (2005). Surf. Sci. Rep..

[cit46] Suresh S. (2001). Science.

[cit47] Domke J., Radmacher M. (1998). Langmuir.

[cit48] Shulha H., Zhai X., Tsukruk V. V. (2003). Macromolecules.

[cit49] Doerner M. F., Nix W. D. (1986). J. Mater. Res..

[cit50] Czajor J., Abuillan W., Nguyen D. V., Heidebrecht C., Mondarte E. A., Konovalov O. V., Hayashi T., Felder-Flesch D., Kaufmann S., Tanaka M. (2021). RSC Adv..

[cit51] Cartagena A., Raman A. (2014). Biophys. J..

[cit52] Schneck E., Papp-Szabo E., Quinn B. E., Konovalov O. V., Beveridge T. J., Pink D. A., Tanaka M. (2009). J. R. Soc., Interface.

[cit53] Ahrens H., Förster S., Helm C. A. (1998). Phys. Rev. Lett..

[cit54] Yamamoto A., Hayashi K., Sumiya A., Weissenfeld F., Hinatsu S., Abuillan W., Nakahata M., Tanaka M. (2022). Front. Soft Matter.

