# Supplementary material for: Ion-specific nanoscale compaction of cysteine-modified poly(acrylic acid) brushes revealed by 3D scanning force microscopy with frequency modulation detection
Source: Nanoscale Adv. 2022 Sep 15;4(23):5027–36. doi: 10.1039/d2na00350c (PMC9680925; doi:10.1039/d2na00350c)
Supplement: NA-004-D2NA00350C-s001 [file NA-004-D2NA00350C-s001.pdf]

Electronic Supplementary Material (ESI) for Nanoscale Advances.
This journal is © The Royal Society of Chemistry 2022

Supplementary information to:

Ion-specific nanoscale compaction of cysteine-modified poly (acrylic acid) brushes revealed by 3D scanning force microscopy with frequency modulation detection

Akihisa Yamamoto*[a], Takahiko Ikarashi[b], Takeshi Fukuma[b, c], Ryo Suzuki[a], Masaki Nakahata[d, e], Kazuki Miyata*[b, c] and Motomu Tanaka*[a, f]

a. Center for Integrative Medicine and Physics, Institute for Advanced Study, Kyoto University, Kyoto 606-8501, Japan

b. Division of Nano Life Science, Kanazawa University, Kanazawa 920-1192, Japan

c. Nano Life Science Institute (WPI-NanoLSI) and Faculty of Frontier Engineering, Kanazawa University, Kanazawa 920-1192, Japan.

d. Department of Materials Engineering Science, Graduate School of Engineering Science, Osaka University, Osaka 560-8531, Japan

e. Department of Macromolecular Science, Graduate School of Science, Osaka University, Osaka 560-0043, Japan

f. Physical Chemistry of Biosystems, Institute of Physical Chemistry, Heidelberg University, 69120 Heidelberg, Germany

* E-mails: yamamoto.akihisa.6w@kyoto-u.ac.jp (AY), k-miyata@staff.kanazawa-u.ac.jp (KM), tanaka@uni-heidelberg.de (MT)

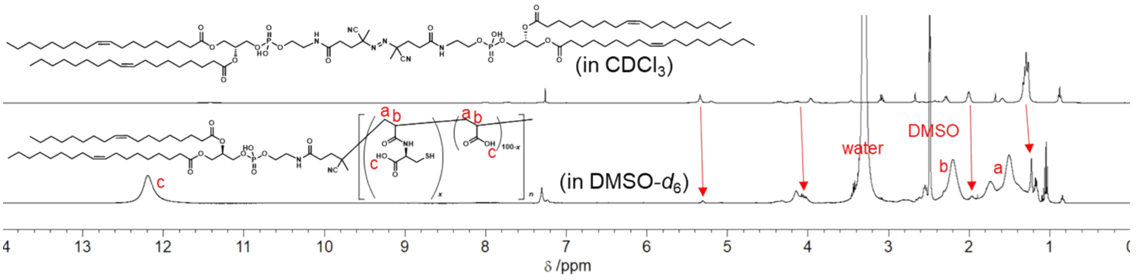

Figure S1. ^{1}H-NMR spectra of ACVA-DOPE (in $CDCl_3$, 30 °C) and DOPE-pAA-Cys5 (in DMSO-d_6, 30 °C).

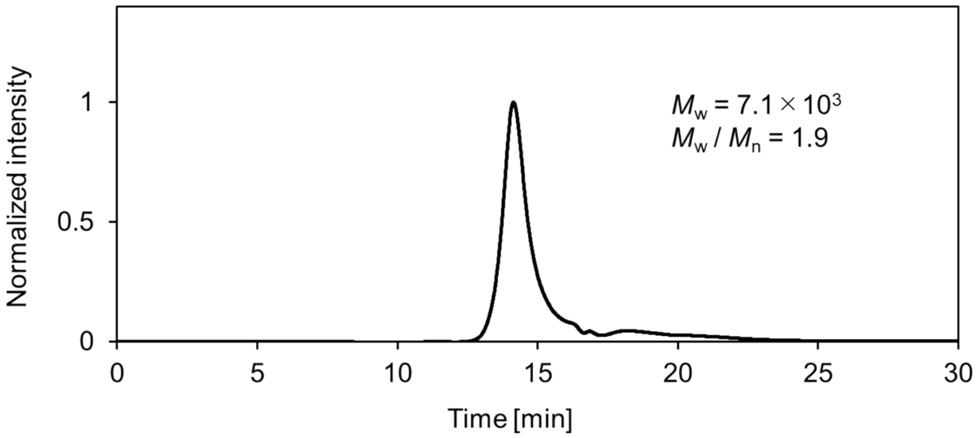

Figure S2. GPC trace of DOPE-pAA-Cys5 (in 10 mM Tris-HCl buffer (pH 7.4) + 100 mM NaCl).

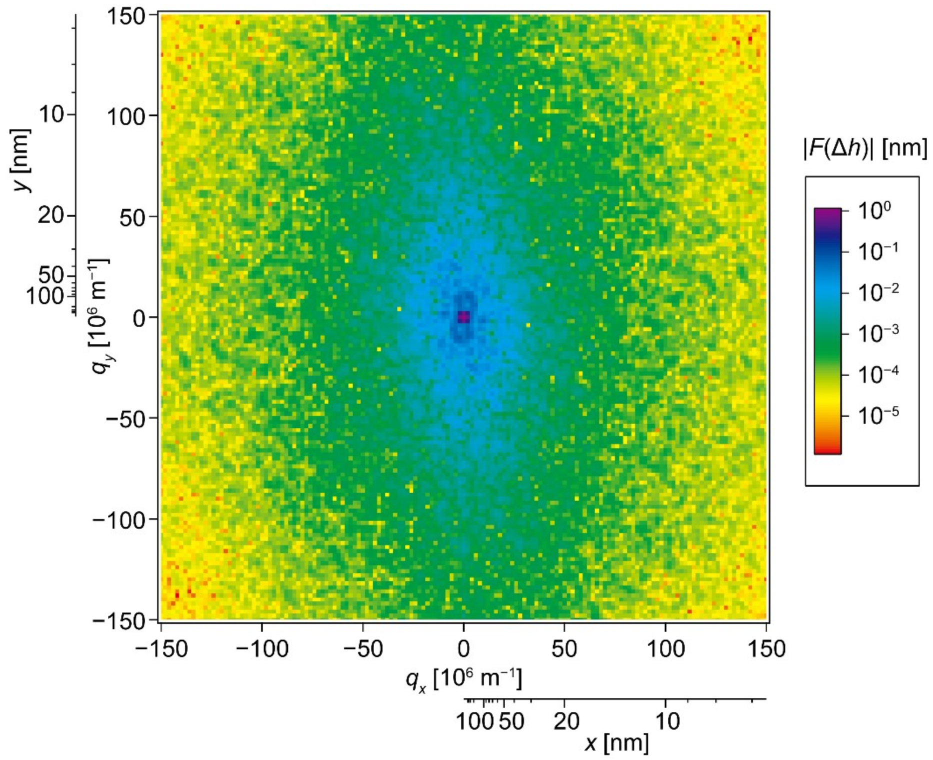

Figure S3. The FFT pattern of topographic profile $|F(\Delta h)|$ in the presence of 1 mM Cd^{2+} ions. The absence of the grid pattern around the center of the FFT profile indicates that there is no ordered lattice alignment of the protrusions.

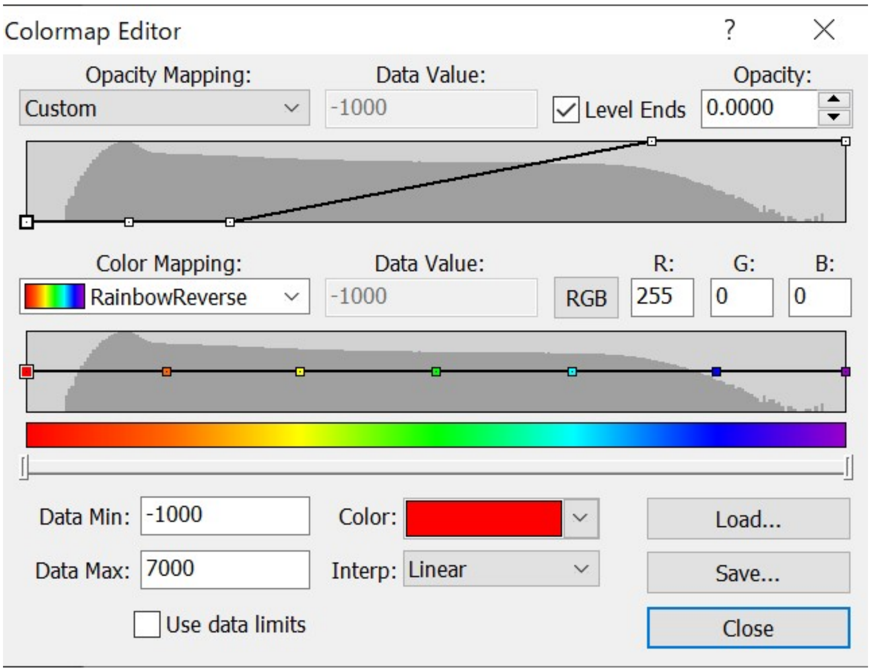

Figure S4. A transparency filter setting applied to the 3D *Δf* maps presented in Figure 3a–3c (in Main text) which were visualized by Voxler 3 (Golden Software, Golden, CO, USA). The transparency filter was adjusted in a manner that the *Δf* distribution at the bulk regions becomes transparent.

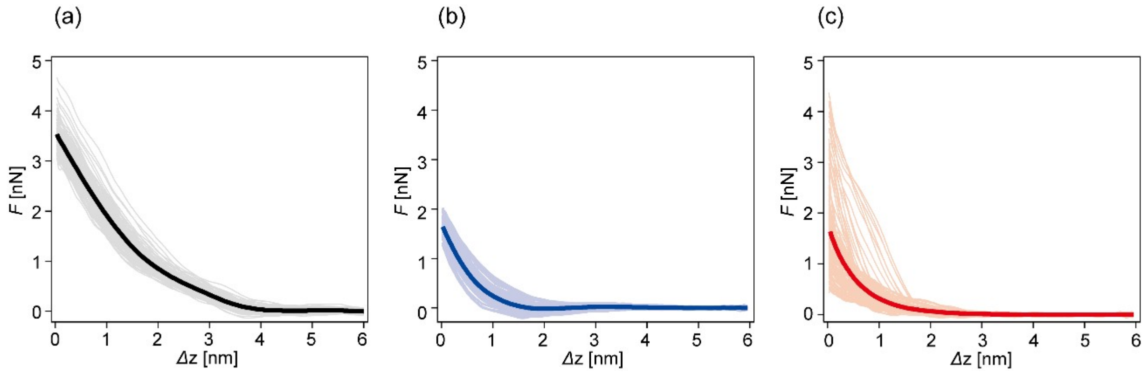

Figure S5. Force curves extracted from the individual *Δf*–*Δz* curves (thin lines) plotted in Figure 4g–4i (in Main text), and its averages (thick lines). (a) 100 mM NaCl, (b) 100 mM NaCl + 1 mM $CaCl_2$, and (c) 100 mM NaCl + 1 mM $CdCl_2$.
